# Low- and high-dose-rate radiation exposure alters the cellular composition and dynamics of the rat mammary epithelium for weeks preceding carcinogenesis

**DOI:** 10.1093/jrr/rraf073

**Published:** 2026-01-13

**Authors:** Kento Nagata, Yukiko Nishimura-Yano, Mayumi Nishimura, Kazuhiro Daino, Daisuke Iizuka, Keiji Suzuki, Shizuko Kakinuma, Tatsuhiko Imaoka

**Affiliations:** Department of Radiation Effects Research, Institute for Radiological Science, National Institutes for Quantum Science and Technology, 4-9-1 Anagawa, Inage-ku, Chiba 263-8555, Japan; Institute for Quantum Life Science, National Institutes for Quantum Science and Technology, 4-9-1 Anagawa, Inage-ku, Chiba 263-8555, Japan; Department of Radiation Effects Research, Institute for Radiological Science, National Institutes for Quantum Science and Technology, 4-9-1 Anagawa, Inage-ku, Chiba 263-8555, Japan; Department of Radiation Effects Research, Institute for Radiological Science, National Institutes for Quantum Science and Technology, 4-9-1 Anagawa, Inage-ku, Chiba 263-8555, Japan; Department of Radiation Effects Research, Institute for Radiological Science, National Institutes for Quantum Science and Technology, 4-9-1 Anagawa, Inage-ku, Chiba 263-8555, Japan; Institute for Quantum Life Science, National Institutes for Quantum Science and Technology, 4-9-1 Anagawa, Inage-ku, Chiba 263-8555, Japan; Department of Radiation Effects Research, Institute for Radiological Science, National Institutes for Quantum Science and Technology, 4-9-1 Anagawa, Inage-ku, Chiba 263-8555, Japan; Institute for Quantum Life Science, National Institutes for Quantum Science and Technology, 4-9-1 Anagawa, Inage-ku, Chiba 263-8555, Japan; Department of Radiation Medical Sciences, Atomic Bomb Disease Institute, Nagasaki University, 1-12-4 Sakamoto, Nagasaki 852-8523, Japan; Department of Radiation Effects Research, Institute for Radiological Science, National Institutes for Quantum Science and Technology, 4-9-1 Anagawa, Inage-ku, Chiba 263-8555, Japan; Department of Radiobiology, Institute for Environmental Sciences, 1-7 Aza-Ienomae, Oaza-Obuchi, Rokkasho-mura, Kamikita-gun, Aomori 039-3212, Japan; Department of Radiation Effects Research, Institute for Radiological Science, National Institutes for Quantum Science and Technology, 4-9-1 Anagawa, Inage-ku, Chiba 263-8555, Japan; Institute for Quantum Life Science, National Institutes for Quantum Science and Technology, 4-9-1 Anagawa, Inage-ku, Chiba 263-8555, Japan

**Keywords:** animal model, dose-rate effect, mammary gland, rat, tissue dynamics

## Abstract

In animals, low-dose-rate radiation induces cancer at a reduced rate compared with a high-dose-rate at an identical cumulative dose, although the underlying mechanism is not well understood. The immediate responses of cells to irradiation are well established, including DNA double-strand break repair, cell-cycle arrest and cell death; conversely, the changes in tissues weeks after irradiation are not well understood. We therefore analysed cellular dynamics in rat mammary tissue weeks after high- or low-dose-rate irradiation. We irradiated 5-week-old rats with 2 Gy (30 Gy/h) or 3- to 5-week-old rats with continuous 2 Gy (6 mGy/h). For histological analysis, luminal cells were identified with anti-cytokeratin (CK) 8 + 18; CK8 + 18^Low^ cells are luminal progenitor cells, and CK8 + 18^High^ cells are luminal mature cells. To evaluate cell composition by flow cytometry, epithelial cells were isolated from mammary tissue. The proliferative potential of luminal progenitor cells—as measured by Ki-67 on paraffin sections—decreased 2 weeks after irradiation at either the high- or low-dose rate but recovered to the control level by 4 weeks. No significant difference was observed in the S phase and total cell-cycle length identified by 5-ethynyl-2′-deoxyuridine and 5-bromo-2′-deoxyuridine or cell death marked by cleaved caspase-3 among the dose-rates. Furthermore, the composition of luminal mature cells changed 2–6 weeks after completing the high- and, to a lesser extent, low-dose-rate radiation exposure, indicating potential proliferative stimulation of luminal progenitor cells related to susceptibility to carcinogenesis. These findings suggest that the altered cell composition and dynamics of luminal cells for several weeks contribute to carcinogenesis.

## INTRODUCTION

Breast cancer is one of the most common cancers in women [1], and it is well established that the mammary gland is highly sensitive to radiation [2]. The risk of cancer development (including breast cancer) increases after radiation exposure, as revealed by studies of atomic-bomb survivors [3, 4]. Epidemiological studies on cohorts of residents in areas of high natural radiation exposure have reported that chronic exposure to very low dose rates does not increase the risk of cancer, suggesting that cancer risk may vary with dose rate when compared to an equivalent cumulative dose [5]. In contrast, associations have been reported between radiation dose and cancer risks from low-dose exposure to occupational radiation [6] or protracted low-to-moderate-dose exposure to environmental radiation [7]. Studies of gene-expression changes after low-dose-rate irradiation have shown that different genes are activated depending on dose rate, time after exposure and tissue type [8]. A significant inflection point in mutation frequency was found between 11 and 21.6 mGy/day in research on tritium exposure [9]. Even with the same dose, long-term low-dose-rate exposure has a smaller effect than acute exposure. This is because, with acute exposure, DNA damage occurs all at once, thereby confounding the accuracy and completion of repair; with long-term low-dose-rate exposure, however, DNA repair occurs before damage accumulates.

Radiation is one of the external factors that cause cancer, like alcohol and tobacco. One of the major cellular responses to radiation-induced DNA damage is the induction of various DNA repair–related signaling pathways, including non-homologous end joining and homologous recombination [10]. DNA double-strand breaks increase in a dose-dependent manner, and insufficient repair of breaks leads to mutations, genomic instability and chromosomal aberrations [11, 12]. Cancer is thought to originate from tissue stem cells that have undergone genetic mutations [13]. Normally, abnormal cells are eliminated by the immune system, but if they escape this process and grow, cancer will develop [14]. The cell-cycle checkpoint mechanism prevents damaged DNA from being replicated or distributed and remaining in abnormal cells. However, a high dose of radiation that causes a large amount of DNA damage over a short period can induce apoptosis or other forms of cell death, which eliminates abnormal cells [15]. Because it has been repeatedly observed that exposure of normal tissues to radiation can stimulate the proliferation rate of transplanted or spontaneously arising tumor cells, worsen malignant phenotypes, and finally kill the host earlier than usual, radiation exposure seems most likely to promote a proinflammatory tissular environment that favors the proliferation of spontaneously arising tumor cells [16].

The mammary gland has a complex, branched tubular structure. During puberty, the mammary gland contains terminal end buds (TEBs) at the leading edge, followed by the duct. Each TEB contains many proliferating progenitor cells and is composed of a single outer layer of basal cells and several inner layers of luminal cells [17]. Induction of female rat mammary carcinogenesis by continuous γ-ray exposure is age-dependent, with a threshold between 24 and 60 mGy/h and an increased risk of carcinogenesis at dose rates >60 mGy/h [18]. The high susceptibility to carcinogenesis that has been demonstrated in young virgin rats is attributable to the presence of a large proliferative compartment, mainly in TEBs and ducts as assessed by pulse-chase experiments with ^3^H-labeled thymidine [19]. The number of TEBs increases during puberty but decreases with the development of the mature mammary-gland structure [20]. In rats, a large number of undifferentiated cells are present in the TEB aged between 3 and 5 weeks. At 7–8 weeks of age, TEBs are rich in proliferating cells and serve as precursors to future mammary ducts. The number of TEBs begins to decline around 9–10 weeks of age, and by 11 weeks, only a few remain [21]. TEBs disappear from the tissue after puberty when mammary tissue is completely formed [17]. In the initial (1–24 h) response after acute radiation exposure, the number of DNA double-strand breaks in luminal cells is higher than in basal cells [22, 23], and basal cells proliferate at a higher rate with a shorter cell cycle compared with luminal progenitor cells [23], suggesting that susceptibility to radiation-induced carcinogenesis may vary among mammary epithelial cells.

Here we documented changes in tissue kinetics related to the dose rate of radiation exposure. We previously reported an association between increased mammary-cancer incidence and acute exposure at a dose rate of 30 Gy/h but not with chronic exposure at 6 mGy/h [18, 24]. We irradiated rats at these two dose rates and evaluated the dynamics of tissue alteration over both the medium- and long-term following radiation exposure in normal mammary tissues to identify factors that contribute to dose-rate effects on carcinogenesis.

## MATERIALS AND METHODS

### Animals

**Fig. 1:**
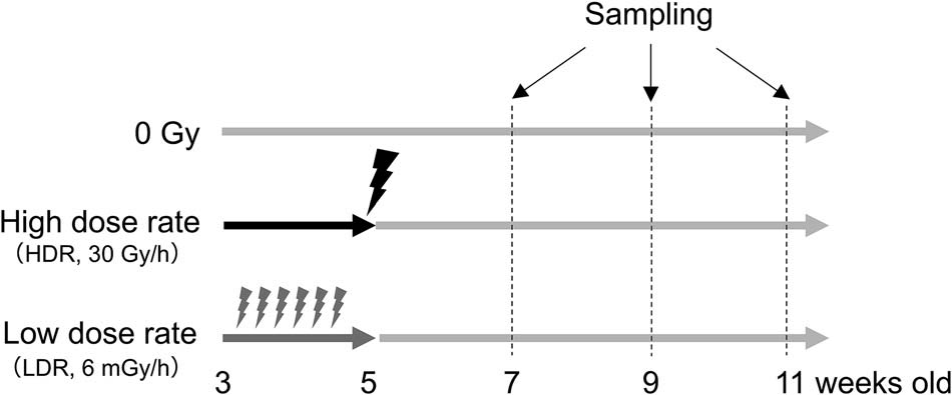
Overview of irradiation experiments. Definitions: 0 Gy, reared in normal cages; high-dose-rate irradiation group, 2 Gy of γ radiation at a dose rate of 30 Gy/h at age 5 weeks; low-dose-rate irradiation group, irradiated with γ rays at a dose rate of 6 mGy/h at age 3–5 weeks to a cumulative dose of 2 Gy. Rats were sacrificed and tissue samples were collected at age 7, 9 or 11 weeks.

We purchased pups of Sprague–Dawley (Jcl:SD) rats (CLEA Japan, Tokyo, Japan) with the mother attached for suckling at age 2 weeks. Five-week-old rats were whole-body irradiated with 2 Gy of ^137^Cs γ-rays using a Gamma cell 40 irradiator (Nordion, Ottawa, Canada, dose-rate, 30 Gy/h) between 9:00 and 12:00 a.m., 3-week-old rats were irradiated continuously for 2 weeks (i.e. until they reached 5 weeks of age) with 2 Gy of ^137^Cs γ-rays at the dose rate of 6 mGy/h, or they were left unirradiated (Fig. 1). Vaginal smears were taken daily over at least one estrous cycle to confirm their cycling before autopsy or injection of labeling chemicals (see below). All rats were autopsied during estrus. All rats were fed a CE-2 diet (CLEA Japan) and provided chlorinated, acidified water ad libitum. They were maintained under a specific pathogen-free condition on a 12-h light/12-h dark cycle. All animal experiments were approved by the Institutional Animal Care and Use Committee of the National Institutes for Quantum Science and Technology (QST) (approval number 20–1008).

### Labeling S-phase cells

To label S-phase cells, 5-ethynyl-2′-deoxyuridine (EdU, Thermo Fisher Scientific) or 5-bromo-2′-deoxyuridine (BrdU, Thermo Fisher Scientific) was dissolved in saline solution to a concentration of 5 mg/ml. Rats in estrus were administered EdU intraperitoneally, and 2 h later, BrdU was administered intraperitoneally, both at 50 mg/kg body weight. Rats were euthanized (see below) 2 h after BrdU administration, and mammary tissue was removed (Fig. 3A).

### Preparation of histological sections

The rats were euthanized by exsanguination under isoflurane-mediated deep anesthesia (4% in air). The fourth mammary gland of 7-, 9- or 11-week-old rats was then collected, extended on a glass slide, and fixed in 10% phosphate-buffered formalin. Paraffin-embedded sections (3 μm) were prepared using a microtome (Leica Microsystems GmbH, Wetzlar, Germany) and deparaffinized in xylene, rehydrated in graded alcohol, and then subjected to antigen retrieval by autoclaving at 95°C in 10 mM Tris–HCl buffer (pH 9.0) for 40 min. These sections were further treated with a cocktail of primary antibodies in a blocking solution (Protein Block, Agilent Technology, Santa Clara, CA, USA) at 37°C for 2 h (Table 1). Thereafter, the sections were rinsed three times in Tris-buffered saline containing 0.05% Tween 20 (50 mM Tris HCl, pH 7.6; TBS-T) and then treated with a cocktail of secondary antibodies at room temperature for 1 h (Table 1). Sections were then rinsed three times in TBS-T. The Click-iT EdU Cell Proliferation kit (Thermo Fisher Scientific, USA) was used for EdU staining. Finally, the sections were again rinsed three times in TBS-T and mounted with Vectashield mounting medium containing 4′,6-diamidino-2-phenylindole (DAPI) (Vector Laboratories, Burlingame, CA, USA).

**Table 1:** Antibody list

Antigen	Clone	Label	Supplier	Catalog number	Species	Dilution	
*Primary antibody*							
CK14	LL002	None	Abcam	ab7800	Mouse	1:500	
CK8 + 18	Polyclonal	None	Progen	GP11	Guinea pig	1:500	
Ki-67	SP6	None	Invitrogen	MA5–14520	Rabbit	1:100	
BrdU		None	Abcam	ab152095	Rabbit	1:100	
Cleaved caspase 3		None	Cell Signaling	9661	Rabbit	1:200	
*Secondary antibody*							
Rabbit IgG	Polyclonal	AF594	Abcam	ab150088	Goat	1:500	
Mouse IgG	Polyclonal	AF488	Abcam	ab150117	Goat	1:500	
Guinea pig IgG	Polyclonal	AF647	Abcam	ab150187	Goat	1:500	
*For flow cytometry*							
CD49f	Mab-5A	FITC	AbD	MCA2034F	Mouse	1:10	
CD24	HIS50	PE	BD	562104PE	Mouse	1:10	
CD31	TLD-3A12	PE/Cy7	eBioscience	25–0310-80	Mouse	1:100	
CD45	OX-1	PE/Cy7	BioLegend	202 213	Mouse	1:100	
Isotype IgG1		FITC	AbD	MCA928F	Mouse	1:10	
Isotype IgM	G155–228	PE	BD	555 584	Mouse	1:10	
Isotype IgG1	P3.6.2.8.1	PE/Cy7	eBioscience	25–4714	Mouse	1:10	

AF, Alexa Fluor; IgG, immunoglobulin G.

### Calculation of the cell-cycle length

To determine the cell-cycle length of epithelial cells, we used an immunohistochemical staining technique [25–27]. Here, ${T}_S$ denotes the length of the S phase of the cell cycle (h), and ${T}_i$ is the interval between the administration of EdU and BrdU. In this study, EdU was administered first and BrdU was administered 2 h later, so ${T}_i$ was set at 2 h. ${P}_{E^{+}{B}^{-}}$ indicates the percentage of cells that were simultaneously EdU-positive and BrdU-negative. ${P}_{E^{+}}$ indicates the percentage of EdU-positive cells. ${T}_S$was determined using the following formula based on a previous report [26].


$$ {T}_S=\frac{T_i}{P_{E^{+}{B}^{-}}/{P}_{E^{+}}} $$

The term ${T}_c$ denotes the total cell-cycle length (h), and ${P}_{Ki-{67}^{+}}$is the percentage of Ki-67-positive cells. The value of the Ki-67 labeling index was taken from the percentage of Ki-67-positive cells determined by immunostaining.


$$ {T}_C=\frac{T_S}{P_{E^{+}}/{P}_{\mathrm{Ki}-{67}^{+}}} $$

### Cell counting

Fluorescence images were obtained using a Disk Scanning Unit Confocal Microscopy system (Olympus, Tokyo, Japan) mounted on an IX83 inverted microscope (Olympus). Cells were counted manually.

### Cell preparation and flow cytometry

Mammary glands were removed from irradiated or non-irradiated rats, cut into small pieces, digested with 0.1% type III collagenase in Hank’s balanced salt solution (HBSS) at 37°C for 3 h with gentle agitation, and washed with HBSS containing 2% fetal bovine serum (FBS) and phosphate-buffered saline (PBS); then, the tissue pieces that did not pass through the 20-μm pore mesh were collected. Tissue pieces were then digested with trypsin in 0.25% ethylenediaminetetraacetic acid solution for 3 min and then 5 mg/mL dispase solution for 1 min at 4°C. Isolated cells that passed through each of a 40 and 10-μm pore diameter mesh were then suspended in 1% FBS in HBSS to achieve a cell concentration of 1 to 2 × 10^6^ cells/mL. The cells were then washed twice with HBSS, suspended in 1 mL HBSS, and 1 μL of dead cell stain (LIVE/DEAD™ Fixable Near-IR Dead Cell Stain kit, for 633 or 635 nm excitation, Thermo Fisher Scientific) was added. After centrifugation, cells were fixed in 5% phosphate-buffered formalin and stored at −80°C. For experimentation, frozen cells were completely thawed at room temperature, transferred to 96-well V-bottoms in the required volume (1 × 10^5^ cells/well), and centrifuged at 300 × *g* for 5 min at 4°C. After aspirating the supernatant, the precipitated cells were resuspended in 200 μL of 1% FBS in PBS and blocked for 30 min. After centrifugation, the supernatant was aspirated, and the cells were suspended in 200 μl of 1% FBS in PBS containing the antibodies (Table 1) and incubated for 30 min on ice. After centrifugation, the supernatant was aspirated, and 200 μL of 1% FBS in PBS was added, and cells were centrifuged again. After a final centrifugation, the precipitated cells were resuspended in 1% FBS in PBS and subjected to analysis with a benchtop flow cytometer (Guava easyCyte system; Merck-Millipore). Data were analysed using FlowJo (Becton Dickinson Japan).

### Statistical analysis

The statistical analysis was performed using R software (version 4.4.1).

## RESULTS

### Cell proliferation after radiation exposure

**Fig. 2:**
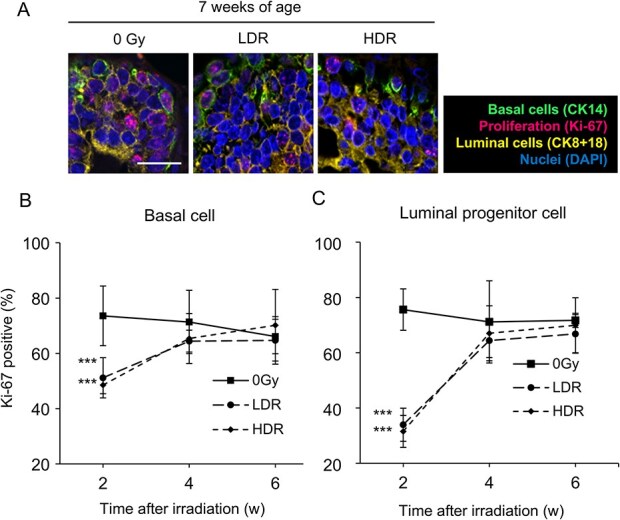
Temporary decrease in the percentage of proliferating cells in the weeks after radiation exposure. A. Identification of proliferating cells in TEBs using anti-Ki-67. Scale bar, 20 μm. B and C. Quantitative analysis of Ki-67-positive basal cells (B) and luminal progenitor cells (C) in rat mammary tissue. LDR, low dose rate; HDR, high dose rate; w, weeks after exposure. ^***^P < 0.001, Tukey’s test after one-way analysis of variance. Vertical bars, SD.

We previously showed that radiation exposure induces DNA double-strand breaks more so in luminal progenitor and mature cells than basal cells of TEBs of the pubertal rat mammary gland [22], although the mid-term alterations during the weeks following the repair of DNA breaks remained to be elucidated. On the other hand, previous studies have shown that ductal hyperplasia or malignant tumors start to appear at 10 to 15 weeks of age in animals exposed to high-dose-rate radiation at younger ages [28, 29]. In this study, to investigate the involvement of tissue dynamics before carcinogenesis occurring within several weeks after radiation exposure, female rats were irradiated at 3 to 5 weeks of age and dissected at 7, 9 and 11 weeks of age (Fig. 1). The TEB of the mammary gland contributes to the elongation of the ductal epithelium by cell proliferation [17]. As the proliferation of cells in the TEB is related to susceptibility of carcinogenesis [30], the fraction of proliferating cells was examined using a cell-proliferation marker, namely an antibody specific for Ki-67, on paraffin sections focused on TEBs, where proliferating cells were abundant. We previously reported that, on paraffin sections of mammary tissue, basal cells could be identified by anti-CK14 and luminal cells by anti-CK8 + 18 [22]. Furthermore, based on differences in CK8 + 18 staining, luminal cells can be divided into luminal progenitor cells and luminal mature cells; CK8 + 18 weakly positive cells are luminal progenitor cells, and CK8 + 18 strongly positive cells are luminal mature cells. Despite the difference in dose rate, 2 weeks after irradiation with 2 Gy (i.e. age 7 weeks), the percentage of Ki-67-positive cells was significantly reduced among basal cells and luminal progenitor cells in the TEB region in high- and low-dose-rate irradiated groups compared with the unirradiated group (Fig. 2A and B). In contrast, 4 and 6 weeks after irradiation (i.e. ages 9 and 11 weeks), the percentage of Ki-67-positive cells of the irradiated groups recovered to the same level as in the non-irradiated group, and there was no significant difference between groups (Fig. 2A and B). These data demonstrated that irradiation suppresses cell proliferation in the TEB region of the mammary gland, although this effect was only transient, with cell proliferation returning to levels comparable to those observed in non-irradiated cells by 4 weeks after exposure.

### Cell-cycle length after radiation exposure

**Fig. 3:**
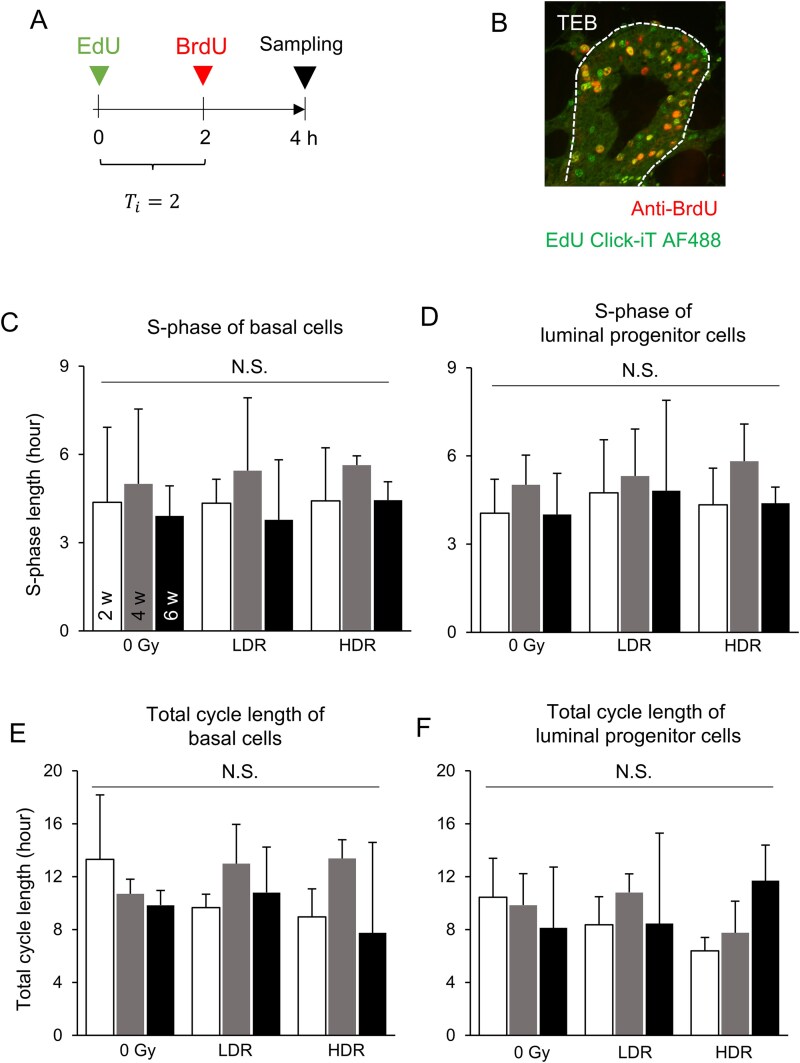
Analysis of cell-cycle length in rat mammary epithelial cells in the weeks after radiation exposure. ​A. Schedule for irradiation experiments and administration of labeling reagents. T_*i*_, interval time (h). B. Labeling of S-phase cells in TEBs. The dashed line outlines the epithelial tissue. C, D. Length of S phase of basal cells (C) and luminal progenitor cells (D). E, F. Total cell-cycle length of basal cells (E) and luminal progenitor cells (F). ​LDR, low dose rate; HDR, high dose rate; w, weeks after exposure. N.S., not significant. Vertical bars, SD.

The above results demonstrated the dynamic changes in cell proliferation in TEBs in response to irradiation. Because the cell-cycle length of cells comprising TEBs is regulated during hormone-related mammary gland development [19], it is possible that the regulation of the cell-cycle length caused these changes observed here. Thus, we investigated whether these alterations were related to changes in cell-cycle length. To assess the S-phase length and total cell-cycle length, we labeled S-phase cells sequentially using two different labels, namely EdU and BrdU, with a 2-h interval; then, fluorescence immunostaining of BrdU and chemical staining to identify EdU were performed simultaneously (Fig. 3A and B). The S-phase length was calculated based on the difference of two labels, and the total cell-cycle length was calculated based on the labeling index and the S-phase length (see Materials and Methods for details). We did not find statistically significant differences in the S-phase length and total cell-cycle length in epithelial cells of the mammary tissue after exposure at either dose rate (Fig. 3C–F). Thus, the cell cycle was not drastically altered by exposure to 2 Gy of radiation in the TEBs.

### Apoptotic cell death after radiation exposure

**Fig. 4:**
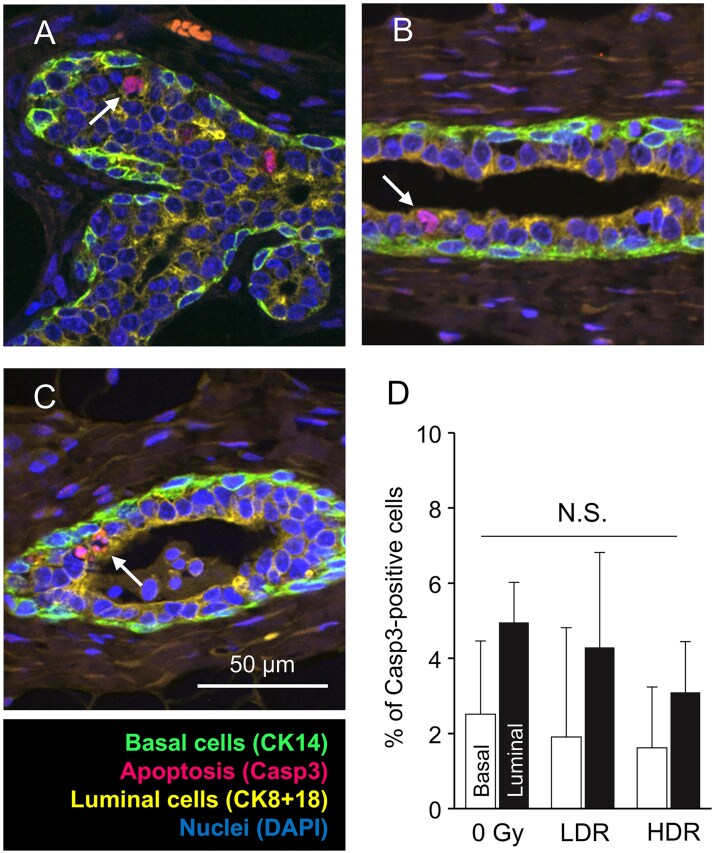
Cell death in mammary tissue 2 weeks after radiation exposure. A–C. Staining of dead cells with anti-cleaved caspase-3 (Casp3) in the 0 Gy group (A), low-dose-rate (LDR) group (B) and high-dose-rate (HDR) group (C). Arrows, Casp3-positive cells. Scale bar in panel C also applies to panels A and B. D. Percentage of Casp3-positive cells after radiation exposure. ​N.S., not significant. Vertical bars, SD.

Apoptosis is rare in the mammary gland but may provide a mechanism of cellular turnover [31]. Thus, the temporal decrease in proliferating cells after irradiation may involve changes in apoptosis. To clarify this, fluorescence immunostaining with cleaved caspase 3, a marker of apoptosis, was performed on apoptotic cells in tissue at 2 weeks post-irradiation (Fig. 4A–C). The number of apoptotic cells in the irradiated group was similar to that in the non-irradiated group, and no significant differences were observed between the two groups (Fig. 4D). Thus, apoptosis was not induced in mammary tissue 2 weeks after radiation exposure.

### Cell composition after radiation exposure

**Fig. 5:**
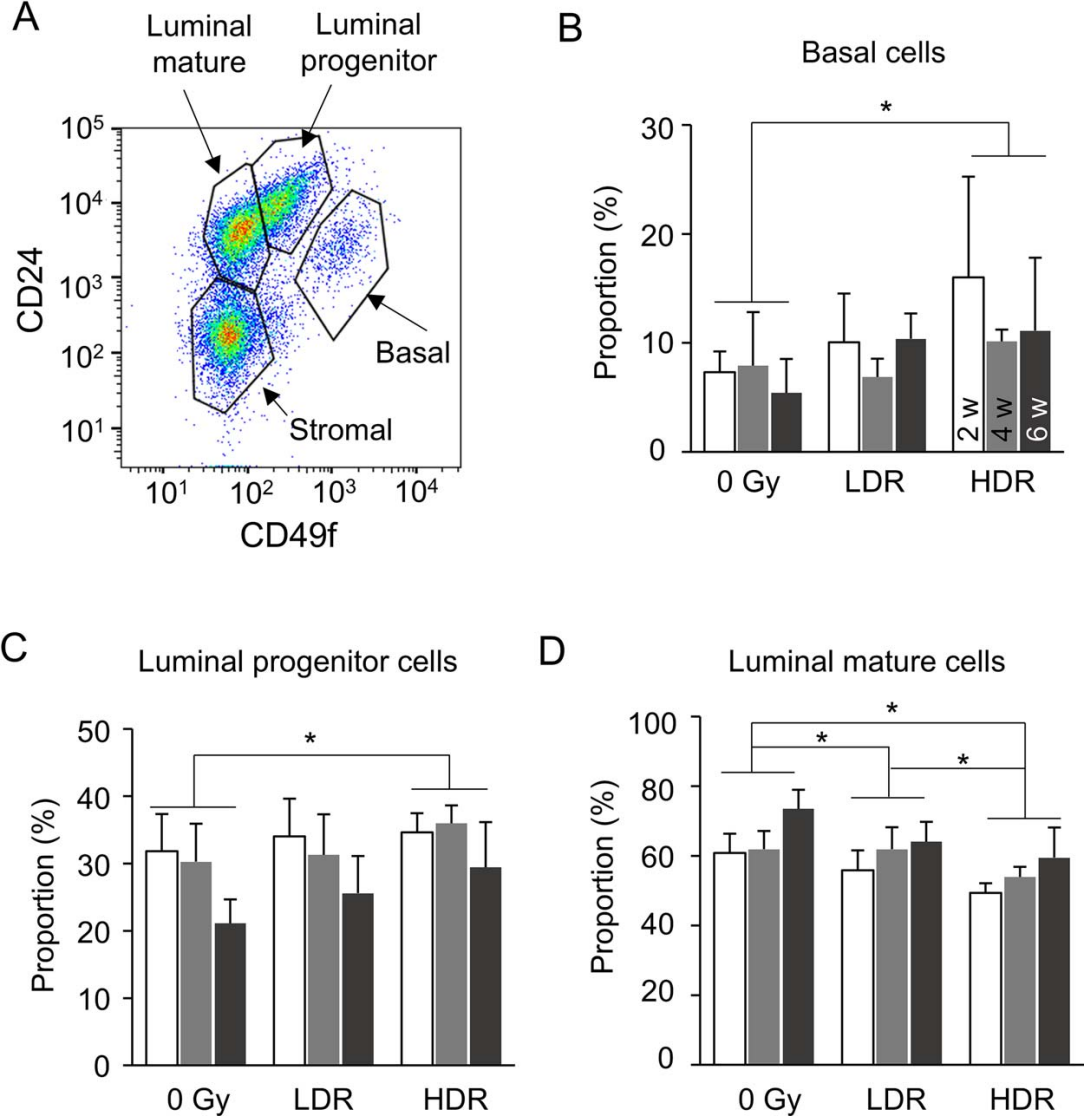
Changes in the percentage of mammary epithelial cells in the weeks after radiation exposure. A. Identification of cell types among the non-irradiated cells by flow cytometry. B–D. Percentage composition of epithelial cells (B, basal cells; C, luminal progenitor cells; D, luminal mature cells) after radiation exposure. ^*^P < 0.05, two-way analysis of variance. ​LDR, low dose rate; HDR, high dose rate; w, weeks after exposure. Vertical bars, SD.

The data presented in Fig. 2 indicated that radiation exposure reduced the proliferative potential of TEB progenitor cells transiently for <4 weeks independently of major alterations in the cell-cycle length and apoptotic rate. We supposed that the transient reduction in progenitor activity, such as cell proliferation, should lead to continuing changes in the mammary epithelium that developed during the post-irradiation period. We therefore quantified the percentage of basal, luminal progenitor and luminal mature cells, which make up the entire mammary epithelium, using flow cytometry with cell-surface markers [23]. Epithelial cells in the rat mammary gland can be classified according to their expression levels of CD49f and CD24, and we previously defined CD49f^high^CD24^mid^ as the basal cell population, CD49f^mid^CD24^high^ as the luminal progenitor cell population, CD49f^low^CD24^high^ as the luminal mature cell population, and CD49f^low^CD24^low^ as the stromal cell population (Fig. 5A). As the study focused on epithelial cells only, the denominator for the calculation of the percentage of composition was the total number of the three cell types—basal cells, luminal progenitor cells and luminal mature cells. First, the changes in the tissues of the non-irradiated group showed that the proportion of luminal progenitor cells decreased with increasing age (Fig. 5C), whereas the proportion of luminal mature cells increased (Fig. 5D). The proportion of luminal mature cells was significantly reduced in the irradiated group, and the degree of reduction was significantly greater in the high-dose-rate irradiation group than in the low-dose-rate irradiation group, indicating a dose-rate effect of radiation (Fig. 5D). In contrast, the proportion of basal and luminal progenitor cells was significantly increased by irradiation only in the high-dose-rate group (Fig. 5B and C). Thus, radiation altered the composition of epithelial cell types in the entire mammary tissue, an effect that lasted at least for several weeks.

## DISCUSSION

In the present study, we investigated cell dynamics in normal rat mammary tissue 2–6 weeks after radiation exposure, when the initial DNA damage responses—such as cell-cycle arrest and DNA repair—have already been completed [22, 23]. We also assessed the effect of dose rate therein, employing two representative dose rates (30 Gy/h and 6 mGy/h) that resulted in high and low mammary cancer incidence as previously reported [18].

First, we observed a temporary decrease in the percentage of Ki-67-positive proliferating cells in rat mammary tissue at 2 weeks after 2 Gy irradiation, which recovered to the same level as non-irradiated rats thereafter (Fig. 2). In general, radiation triggers activation of DNA-damage checkpoints and causes a transient cell-cycle arrest that continues for a few hours to days, during which DNA damage is repaired [32]. In the present study, the transient suppression of proliferation was observed after 2 weeks of irradiation (Fig. 2B and C) and thus was considered to be distinct from cell-cycle arrest. Similarly, BrdU incorporation experiments in mice and rats after high-dose exposure suggest that the cell cycle is arrested in either G1, S or G2 phase, extending the length of the cell cycle [30, 31]. Because this is a transient event that should end shortly after exposure, our observation of no difference in cell-cycle and S-phase lengths 2 weeks or more after radiation exposure is not contradictory.

Breast-cancer development correlates positively with radiation dose rate [33]. In rats, we previously demonstrated that the induction of mammary carcinogenesis by continuous γ-irradiation is minimal in adults irradiated at 4 Gy at a dose rate of 3–24 mGy/h but dramatically increases at a dose rate of 60 mGy/h or higher [18]. Another study also indicated no significant increase in mammary cancer incidence in female rats chronically exposed to γ rays up to 1.1 Gy at a dose rate of 0.0076 Gy/h [34]. A dose-rate effect in mammary cancer has also been shown in female BALB/c mice [35]. In the present study, the flow cytometric analysis showed a dose-rate-dependent decrease in the percentage of luminal mature cells 2 to 6 weeks after irradiation (Fig. 5D). This result suggests that the altered composition of mammary epithelial cells during the weeks post-exposure is a key factor contributing to the dose-rate effect in carcinogenesis. Luminal progenitor cells in the mammary gland have a very short cell cycle as we observed (Fig. 3). As the short cell-cycle length is a hallmark of cancer susceptibility [36], the skewed cell composition of the mammary epithelium towards increased luminal progenitor cells might provide a microenvironment that is highly susceptible to carcinogenesis.

We used a simplified method for measuring cell-cycle length. Russo et al. [20] evaluated the fraction of proliferative cells in the TEB region and reported that 87%, 78% and 72% of the luminal cells in the TEB were proliferative at ages 40, 55 and 70 days in non-irradiated rats. In our study, the values for the labeling index of luminal progenitor cells (70%–80%, Fig. 2C) were consistent with those reported in the previous study. As reported by Russo et al., T_S_ and T_C_ in the TEB region of young virgin rats (7 weeks old) were estimated to be 7.2 and 11.65 h, respectively [20], although this report did not measure luminal and basal cells separately. Therefore, utilizing cytokeratin-specific antibodies allowed us to precisely differentiate epithelial cell subtypes, thereby enabling the estimation of their respective cell cycle durations (basal cells, T_S_ = 4.37 ± 2.55 h and T_C_ = 13.31 ± 4.88 h; luminal cells, T_S_ = 4.05 ± 1.16 h and T_C_ = 10.45 ± 2.94 h; Fig. 3D and F). In our study, the estrous cycle was standardized, although this information has not been described in previous studies. Furthermore, it should be noted that the standard errors for the T_S_ values in our study were large, so caution should be exercised when considering whether shortening of the S phase actually occurred.

We found no significant increase in apoptotic cell death in the irradiated mammary tissue (Fig. 4). In general, radiation-induced cell death occurs within a few hours [37, 38]. For example, the number of apoptotic cells increases during the period 4–24 h after 4 Gy of γ radiation, and apoptosis is more prevalent in luminal cells than in basal cells [23]. Apoptotic cell death, on the other hand, plays physiological roles in the normal mammary gland. In pubertal mice, some macrophages that localize around the TEBs have apoptotic bodies in their cytoplasm, contributing to ductal elongation by removal of apoptotic cells [39, 40]. Thus, the apoptotic cell death that we observed during the period 2–6 weeks after exposure should be related to these and other physiological processes rather than a remnant of radiation-induced apoptosis. On the other hand, changes in cell proliferation and cell death occur immediately after exposure to radiation, and the possibility that these changes could also subsequently lead to carcinogenesis cannot be ruled out. The early dynamics following radiation exposure were not addressed in this study, because we intended to observe cell dynamics several weeks after radiation exposure, and thus, further investigation is required.

Finally, we analysed the composition of mammary epithelial cells and found that the proportion of basal cells and luminal progenitor cells was increased by acute radiation exposure, whereas the proportion of luminal mature cells decreased in a dose-rate-dependent manner (Fig. 5). To test the possibility that radiation affected cell proliferation to induce these changes, we quantified Ki-67-positive basal and luminal progenitor cells, showing that radiation exposure caused a transient (<4 weeks) decrease in the percentage of proliferating cells, which returned to normal by 4 weeks (Fig. 2). This result supports the idea that the production of luminal mature cells by progenitors is reduced after radiation exposure, leading to altered cell composition. However, because the transient decrease in proliferation was similar at the low- and high-dose rates (Fig. 2), additional factors may be involved in the dose-rate dependency of cell composition.

In this study, analysis using anti-Ki-67, which is a cell proliferation marker, revealed that both basal cells and luminal progenitor cells exhibited a similar proliferation pattern after radiation exposure: a decrease followed by an increase (Fig. 2B). Furthermore, flow cytometry analysis showed that high-dose-rate radiation exposure significantly increased the proportion of basal cells and luminal progenitor cells (Fig. 5B and C). On the other hand, recent studies have reported that luminal mature cells regulate both basal cells and luminal progenitor cells through the growth factors RANKL, WNT4 and AREG [41]. Exposure to radiation may decrease the number of luminal mature cells, which then may reduce the secretion of these growth factors. This might affect both basal cells and luminal progenitor cells and lead to an increase in both types of cells.

We also examined two other potentially important mechanisms known in the mammary gland, namely cell-cycle length and apoptosis. Cell-cycle length was assessed using the dual labeling method (i.e. EdU and BrdU), indicating that the S-phase length and total cell-cycle length did not change in basal or luminal epithelial cells after irradiation (Fig. 3). In addition, there was no change in the prevalence of apoptosis in these epithelial cells 2 weeks after radiation exposure (Fig. 4). These results strongly suggest that radiation exposure induces alterations in cellular composition and dynamics in the rat mammary gland at least for several weeks.

Changes in proliferating cells following exposure to radiation have been reported in other tissues. For example, irradiation promotes atrophy of thymus tissue in mice, yet the number of cells increases during the recovery period [42]. Furthermore, the proportion of proliferating cells in the mice liver increases during the pubertal period [43]. These reports suggest that cell proliferation following radiation exposure may lead to increased risk of cancer development at a later stage [42, 43]. In summary, our observation of an increased number of proliferating cells throughout the mammary tissue due to high-dose-rate exposure (Fig. 5B and C) is similar to that seen in other organs.

Our results reveal previously unnoticed radiation-induced alterations in cellular composition and dynamics of proliferation that last for several weeks. As a result, a microenvironment that promotes the proliferation of luminal cells might be formed within the tissue. This may increase susceptibility to mutations arising from replication stress. Furthermore, cells that carry these mutations may clonally expand and subsequently transform into cancer cells. Because the extent of the changes was reduced by lowering the dose rate, these alterations may provide a mechanism for the dose-rate effect in carcinogenesis.

## PRESENTATION AT CONFERENCES

65^th^ and 66^th^ Annual Meetings of the Japanese Radiation Research Society, 59^th^ Annual Meeting on Radioisotopes and Radiation.
